# Absorption of omega-3 fats from carbohydrate and proteinaceous food matrices before and after storage

**DOI:** 10.1002/fsn3.204

**Published:** 2015-01-22

**Authors:** Tracey J Smith, Ann Barrett, Danielle Anderson, Marques A Wilson, Andrew J Young, Scott J Montain

**Affiliations:** 1 Military Nutrition Division, U.S. Army Research Institute of Environmental MedicineKansas Street, Building 42, Natick, Massachusetts; 2 Performance Optimization Research Team, Combat Feeding Directorate, Natick Soldier, Research and Engineering CenterKansas Street, Building 36, Natick, Massachusetts

**Keywords:** Absorption, encapsulation, fatty acids, omega 3 fatty acids

## Abstract

Development of n-3 fortified, shelf-stable foods is facilitated by encapsulated docosahexaenoic acid (DHA) and eicosapentaenoic acid (EPA), since natural n-3 food sources cannot withstand high temperature and prolonged shelf life. Organoleptic stability of n-3 fortified, shelf-stable foods has been demonstrated, but chemical changes in the food matrix throughout storage could conceivably impact digestibility of the protein-based encapsulant thereby compromising n-3 bioavailability. We assessed the effect of prolonged high-temperature storage and variations in food matrix (proteinaceous or carbohydrate) on the time course and magnitude of blood fatty acids changes associated with ingestion of n-3 fortified foods. Low-protein (i.e., cake) and high-protein (i.e., meat sticks) items were supplemented with 600 mg encapsulated DHA+EPA, and frozen either immediately after production (FRESH) or after 6 months storage at 100°F (STORED). Fourteen volunteers consumed one item per week (randomized) for 4 weeks. Blood samples obtained at baseline, 2, 4, and 6 h post-consumption were analyzed for circulating long-chain omega 3 fatty acids (LCn3). There was no difference in LCn3 area under the curve between items. LCn3 in response to cakes peaked at 2-h (FRESH: 54.0 ± 16.8 *μ*g/mL, +18%; STORED: 53.0 ± 13.2 *μ*g/mL, +20%), while meats peaked at 4-h (FRESH: 51.9 ± 12.5 *μ*g/mL, +22%; STORED: 53.2 ± 16.9 *μ*g/mL, +18%). There were no appreciable differences in time course or magnitude of n-3 appearance in response to storage conditions for either food types. Thus, bioavailability of encapsulated DHA/EPA, within low- and high-protein food items, was not affected by high-temperature shelf-storage. A shelf-stable, low- or high-protein food item with encapsulated DHA/EPA is suitable for use in shelf-stable foods.

## Introduction

Omega-3 fatty acids (n-3s) have purported anti-inflammatory properties and subsequent beneficial effects on human health (Kris-Etherton et al. 2003; Mamalakis et al. 2002; Michael-Titus et al. 2007; Wu et al. 2007). Dietary intake recommendations for n-3s range from 200 mg/day to 1 g/day (A Report of the Panel on Macronutrients 2002), and sources include oily fish and plant-derived sources that are rich in *α*-linolenic acid (ALA), a precursor to long-chain n-3 fatty acids (LCn3). Much of the research on health benefits of n-3s in the diet has focused on cardiac function and reductions in mortality rates associated with cardiovascular disease (CVD), coronary heart disease (CHD), and myocardial infarction (MI) (Kris-Etherton et al. 2003). Beyond cardiac health, some evidence suggests that n-3s reduce psychiatric symptoms such as depression and suicidal ideation (Mamalakis et al. 2002). These polyunsaturated fatty acids (PUFA's) have also been found to have significant therapeutic potential in acute neurological trauma, such as spinal cord and traumatic brain injuries. Further, n-3s, specifically docosahexaenoic acid (DHA), have been shown to have neuroprotective effects and proregenerative potential in reducing the recovery time from these neurological lesions/injuries (Michael-Titus 2007; Wu et al. 2007).

Inadequate dietary intake of n-3s could be a health-risk factor for military personnel. Indeed, n-3 status is lower in military personnel compared to the general U.S. population (Lewis et al. 2011). Santos et al. (2012) reported that supplementation with DHA (360 mg/day) and EPA (540 mg/day) over the course of 4 weeks decreased C-reactive protein (CRP), and attenuated inflammatory activity induced by five consecutive days of intense military training (i.e., constant physical exertion and sleep/food deprivation). Based on the potential health benefits of n-3s for military personnel (Santos et al. 2012), and their documented poor fatty acid profiles (Lewis et al. 2011), supplementing or fortifying the naturally occurring n-3s in military rations might be beneficial.

Adding n-3s to shelf-stable food items like those used for military rations, presents challenges. For example, ration items must meet rigorous shelf-life specifications (i.e., 3 years at 80°F or 6 months at 100°F). Omega 3 fatty acids have a high degree of unsaturation and, therefore, react readily with oxygen, becoming rancid easily during storage (Nawar 1985). Oxidation is accelerated when n-3s are exposed to high temperatures and humidity, that can occur during storage. Food scientists have developed encapsulation techniques, whereby molecules are coated in gelatin (a protein), which prevents degradation of n-3s (Torres-Giner et al. 2010). In ration items fortified with microencapsulated DHA and EPA, the quantity of n-3s (and sensory characteristics of the food items) are maintained after storage under typical shelf-life conditions (Barrett et al. 2014). Pilot testing in our laboratory confirmed that microencapsulated DHA/EPA was absorbed and incorporated into the plasma (∽40% increase from baseline) within 4 h after volunteers consumed pound cake fortified with microencapsulated DHA/EPA (600 mg).

Although the bioavailability of microencapsulated n-3s has been demonstrated in response to consumption of “fresh” food items, the bioavailability of microencapsulated n-3s in foods exposed to high heat and prolonged storage conditions has not been evaluated. Pilot testing in our laboratory (Tracey J. Smith, Ann Barrett, Danielle Anderson, Marques A. Wilson, Andrew J. Young & Scott J. Montain unpubl. data) indicated that there was no appreciable difference in LCn3 plasma absorption after volunteers consumed DHA/EPA-enriched (600 mg) pound cake that was either “fresh” (i.e., frozen after production) or previously stored for 6 months at 100°F (∽40% and 45% increase from baseline, respectively). However, absorption rates may differ if DHA and EPA are incorporated into protein-rich food items (e.g., dehydrated meat sticks). For example, chemical reactions (e.g., disulfide bonding, and Maillard browning) between the gelatin encapsulate and the food protein may strengthen the encapsulate, thus making the encapsulate less susceptible to digestive enzymes (Meade et al. 2005) and, ultimately, reducing the bioavailability of the LCn3. Therefore, this study evaluated plasma n-3 concentrations following consumption of low- and high-protein containing ration items enriched with microencapsulated n-3s, that were either “fresh” (i.e., frozen after production) or previously stored for 6  months at 100°F. We hypothesized that: (1) prolonged storage of a low-protein food item would have no effect on plasma LCn3 concentrations; (2) that food matrix (carbohydrate vs. protein) would have no effect on plasma LCn3 concentrations when products were not subjected to prolonged storage; and (3) that plasma LCn3 concentrations would be higher after ingestion of a “fresh” protein-rich food item or previously stored, low-protein food item enriched with encapsulated DHA/EPA, compared to a previously stored, protein-rich food item enriched with encapsulated n-3s. Data from this study will inform ration developers and food manufacturers as to whether microencapsulated DHA/EPA in a carbohydrate and/or protein food matrix are viable options for inclusion in shelf-stable food items.

## Materials and Methods

### Participants

Participants were military and civilian personnel assigned to Natick Soldier Systems Center, Natick, MA. Complete data were collected on 14 participants. The adequacy of this sample size was estimated, using pilot data from our laboratory and data from Raatz et al. (2009), indicating that 14 participants were required to detect a 50% difference in the area under the curve (AUC) for circulating LCn3 between trials, with a 50% SD of the difference (*α* = 0.01, power = 0.83, *P* < 0.01). Study participation was voluntary. Each volunteer gave their written, informed consent after an oral explanation of the study. The study was approved by the Institutional Review Board, United States Army Research Institute of Environmental Medicine (USARIEM), Natick, MA.

Individuals were included if they were at least 18 years old, generally healthy, not taking medications (including nonsteroidal anti-inflammatory drugs and aspirin), and were not pregnant or lactating. Further, individuals were excluded if they had a history of alcoholism or typically consumed >4 alcoholic beverages daily or 6 on one occasion more than once a month within the past 6 months; consumed ≥1 serving (∽3 oz) of n-3 rich seafood or omega-3 rich eggs per week over the previous past month; or, habitually consumed n-3 dietary supplements. Each participant completed a baseline questionnaire to determine demographic information (age, sex, race, and ethnicity).

### Study design

During four sessions over the course of ∽4 weeks (i.e., 1 session per week), participants were provided with food items in a prospective, randomized, cross-over study. DHA and EPA (600 mg; DSM Nutritional Products LLC, Parsippany, NJ) were incorporated into two different food items varying in protein content (i.e., pound cake and meat sticks, Table1) and storage conditions were manipulated. Nonesterified fish oil was emulsified, spray dried, then microencapsulated in gelatin prior to being incorporated into all ration items. DHA and EPA content of the ration items were chemically determined and food items were packaged in trilaminate (i.e., polyethylene-foil-polyester) pouches. Oxygen scavenger sachets were included with the cakes, while the meat sticks were vacuum packed. Further, all food items underwent microbiological testing by trained microbiologists (Combat Feeding Directorate, NSRDEC, Natick, MA) to ensure that no harmful bacteria or mold was growing in the food items prior to consumption.

**Table 1:** Nutrient content of test meals

	Pound cake	Meat stick
Energy (kcals)	270	218
Carbohydrates (g, %^1^);	74, 55%	8, 15%
Total fat (g, %^1^)	24, 40%	10, 41%
Saturated fat (g)	0	4
Unsaturated fat (g)	6	6
Protein (g, %^1^)	6, 5%	24, 44%

	Fresh^2^	Stored^3^	Fresh^2^	Stored^3^
DHA (mg)^4^	148.3 ± 3.0	147.0 ± 17.4	213.3 ± 14.8	228.5 ± 3.6
EPA (mg)^4^	434.8 ± 24.9	433.3 ± 13.3	410.2 ± 30.9	428.6 ± 12.1

1 Percent of energy from carbohydrate, fat, and protein within each food item.

2 Frozen after production.

3 Stored for 6 months at 100°F and then frozen prior to consumption.

4 Three samples of each food item were analyzed for DHA and EPA content at the end of the data collection period. The mean ± SD is presented.

Plasma n-3 concentrations were assessed after consumption of: (1) a low-protein food item (i.e., pound cake) that was enriched with microencapsulated DHA/EPA (600 mg) and was not subjected to high temperature and prolonged storage (CAKE-FRESH); (2) a low-protein food item that was enriched with microencapsulated DHA/EPA (600 mg) and previously stored for 6 months at 100°F (CAKE-STORED); (3) a high-protein food item (i.e., intermediate moisture meat stick) that was enriched with microencapsulated DHA/EPA (600 mg) and was not subjected to high temperature and prolonged storage (MEAT-FRESH); and, (4) a high-protein food item (that was enriched with microencapsulated EPA/DHA (600 mg) and previously stored for 6 months at 100°F (MEAT-STORED).

### Dietary intake

Measurement of n-3s in circulating blood is sensitive to n-3s in the diet, thus, it was critical to ensure that background n-3 levels remained low and constant when determining the absorption rate of n-3s in response to consumption of n-3 containing food items. Therefore, volunteers were asked to refrain from consuming all seafood, food items enriched with n-3s (e.g., eggs), and n-3 dietary supplements throughout the study. Prior to each trial, trained study staff interviewed volunteers using a short questionnaire to assess compliance with dietary restrictions.

On the morning of each trial, volunteers arrived at the testing site after an overnight fast (∽12 h). Additionally, volunteers were asked to abstain from alcohol in the 48 h prior to each trial. Aside from the DHA/EPA-enriched ration items, volunteers were not allowed to consume any food or beverages (besides water) during each trial. Volunteers were asked to record all foods and beverages that they consumed the day before each trial to identify any potential confounders and were given oral and written instructions to do so. Volunteers were encouraged to record intake at the time the food or beverage was consumed rather than recording at the end of the day. Food records were reviewed and finalized for accuracy by trained study staff, and analyzed for nutrient content using computer-based nutrition analysis software (Food Processor, ESHA Research, Salem, OR).

### Specimen collection and analysis

**Figure 1:**
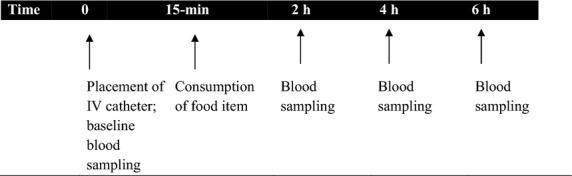
Timing of blood collection.

An indwelling venous catheter was placed in the volunteers’ forearm or antecubital space upon arrival to the testing site. In the event that a catheter failed during the data collection period prior to the final blood draw, a venipuncture was performed. Baseline blood samples were taken after placement of the catheters and at various time points as indicated in Figure1. Blood specimens were collected in EDTA (for plasma) or non-EDTA (for serum) vacutainers or monovettes. Plasma and serum were obtained after spinning blood in a cold and room temperature centrifuge, respectively.

**Figure 2:**
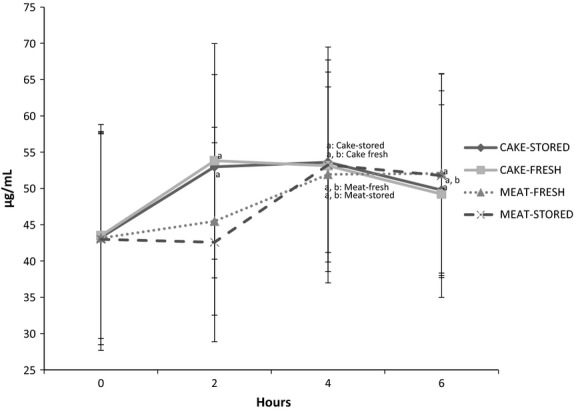
Circulating long-chain omega 3 fatty acid concentrations. (a) Significant within-group difference from 0 h (*P *< 0.05). (b) Significant within-group difference from 2 h (*P *< 0.05).

Plasma was stored in cryotubes at −80°C and shipped on dry ice to the National Institutes of Health for analysis of fatty acids. Blood samples were placed into vials containing 2 mL CHCl3, 1 mL BHT-MeOH, and a known quantity of 23:0 methyl ester as an internal standard. One milliliter of 0.2 mol/L Na2HPO4 was then added after brief vortexing. The samples were then capped under N2 and vortexed again. After centrifugation, CHCl3 was removed and dried under N2. Samples were then methylated with BF3-MeOH for 60 min (Morrison and Smith 1964). Samples were kept cold and under N2 to prevent oxidation. Gas chromatography was then performed using a Hewlett-Packard (HP) 5890 series II with a flame ionization detector, an auto-sampler, and a FFAP capillary column (J&W Scientific, Folsom, CA). The fatty acid peaks were identified using authentic standards (NuCheck Prep, Elysian, MN) and the fatty acids were quantified by comparison to peak areas of the 23:0 internal standard.

Serum was stored in cryotubes at −80°C for analysis of triglycerides, phospholipid, and cholesterol esters (total cholesterol, high-density lipoprotein, and low-density lipoprotein) at USARIEM's central laboratory. Triglycerides (TG) and cholesterol esters were analyzed using the Siemens Dimension Analyzer (Siemens USA, Malvern, PA, USA). Phospholipids were analyzed with a colorimetric assay (EPLP-100) from BioAssay Systems (Hayward, CA, USA). The serum panel was included to consider if baseline lipid profile differences contribute to between-subject variability in plasma n-3 AUC after test meal consumption.

### Calculations and statistical analyses

The dependent variable of interest was n-3 appearance in circulation during the observation period. Area under the Curve (AUC) was calculated individually for each volunteer's four test meals for the following outcome measures: total n-3 polyunsaturated fatty acids (PUFA) and LCn3 (i.e., ≥20 carbons and ≥3 double bonds).

Briefly, “Area Under the Curve with respect to the increase” (AUC_i_) (Pruessner et al. 2003) represents the total AUC for all measurements with consideration for the time difference between each measurement (*t*_*ί*_) and their distance from the first value (i.e., baseline). Data were analyzed using IBM SPSS statistical package version 19.0 (IBM Inc., Armonk, New York) or equivalent. The Shapiro–Wilk test was used to examine normality of each variable. One-way repeated measures analysis of variance was used to compare postprandial n-3 absorption rate in terms of the calculated AUCs. Two-way repeated measures analysis of variance was used to analyze meal by time differences between trials in regards to blood concentrations of n-3s, that is, time (hours) to peak concentration and peak concentrations. If a significant F-ratio was observed for a main effect, post-hoc comparisons were made using Sidak's adjustment for multiple comparisons. Results are presented as mean ± SD. Statistical significance was set a priori at *P* < 0.05.

## Results and Discussion

Complete data were collected on 14 total participants (9 males/5 females; 26 ± 12 years). Biochemical analysis of the food items confirmed that there was no difference in DHA/EPA content at the beginning versus the end of the study (Table1).

### Dietary intake

All volunteers reportedly refrained from consuming all seafood, food items enriched with n-3s (e.g., eggs), and n-3 dietary supplements throughout the study. Additionally, all volunteers reportedly abstained from alcohol in the 48 h prior to each test session; and, consumed only water for the previous 12 h before each test session. There were no significant differences in participants’ dietary intake of fat or n-3s during the 24-h prior to each test session; and, mean (±SD) values across the four trials were 78.4 ± 25.7 g and 0.67 ± 0.30 g, respectively.

### Baseline total cholesterol, triglycerides, and cholesterol esters

There were no significant between trial differences in participants’ baseline total cholesterol, TG, HDL, LDL, and phospholipids; and, mean (±SD) values across the four trials were 169.6 ± 38.9, 78.2 ± 41.8, 62.0 ± 22.8, 96.0 ± 28.2, and 1.6 ± 0.4, respectively. Absorption of n-3s did not covary with baseline total cholesterol, TG, HDL, LDL, or phospholipids, that is, n-3 absorption responses were independent of these baseline measures.

### Fatty acid responses

There were no significant differences in AUC between food items, regardless of storage conditions or food matrix, with respect to total circulating LCn3. However, when LCn3 were analyzed individually, AUC for EPA (but not DPA or DHA) was significantly higher in response to the cakes versus the meat sticks (Table2). There appeared to be differences in time course between food types, and treatment-by-time interactions indicated that plasma LCn3 concentrations significantly increased between baseline and 2 h after eating the cake items (CAKE-FRESH: +18%; CAKE-STORED: +20%, *P* < 0.001), while LCn3 did not significantly increase above baseline until 4 h after eating the meat items (MEAT-FRESH: +22%; MEAT-STORED: +18%, *P* < 0.001). Plasma LCn3 concentrations remained elevated from 2 to 6 h and from 4 to 6 h in response to the cake and meat items, respectively.

**Table 2:** Select omega-3 fatty acid responses (mean ± SD)

		Food item			
		CAKE-FRESH^1^	CAKE-STORED^2^	MEAT-FRESH^1^	MEAT-STORED^2^
Long-chain omega-3 fatty acids (*μ*g/mL)^3^	AUC	45.7 ± 31.2	46.7 ± 49.4	31.1 ± 32.4	28.5 ± 33.4
Total polyunsaturated omega-3 fatty acids (*μ*g/mL)	AUC	82.5 ± 51.1*	78.3 ± 81.9	24.8 ± 35.2	24.2 ± 38.4
EPA (*μ*g/mL)	AUC	42.8 ± 18.2*	44.2 ± 19.3*	24.6 ± 13.8	24.7 ± 13.2
DPA (*μ*g/mL)	AUC	-2.3 ± 13.0	0.86 ± 9.7	2.7 ± 6.5	0.1 ± 7.2
DHA (*μ*g/mL)	AUC	5.2 ± 12.5	1.6 ± 23.6	3.8 ± 15.5	3.7 ± 19.3

AUC, area under the curve; EPA, eicosapentaenoic acid or 20:5 omega 3; DPA, docopentaenoic acid or 22:5 omega 3; DHA, docosahexaenoic acid or 22:6 omega 3.

1 Frozen after production.

2 Stored for 6 months at 100°F and then frozen prior to consumption.

3 Omega-3 fatty acids with ≥20 carbons and ≥3 double bonds (i.e., eicosapentaenoic acid, docopentaenoic acid, and docosahexaenoic acid).

* Indicates significant difference from meat items, *P* < 0.05.

**Figure 3:**
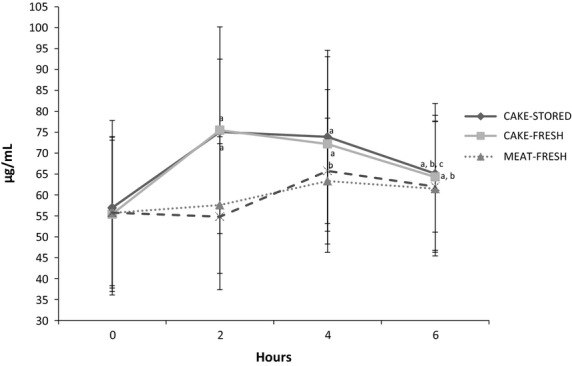
Total circulating n-3 PUFA concentrations. (a) Significant within-group difference from 0 h (*P *< 0.05). (b) Significant within-group difference from 2 h (*P *< 0.05). (c) Significant within-group difference from 4 h (*P *< 0.05).

The total n-3 PUFA AUC (Table2), was significantly greater in response to CAKE-FRESH (82.5 ± 51.5) compared to MEAT-FRESH (25.8 ± 35.2) and MEAT-STORED (25.2 ± 34.5), *P* < 0.01, however, differences in AUC between CAKE-STORED and the meat items did not reach statistical significance. Generally, these results are supported by the fact that plasma total n-3 PUFA concentrations significantly increased from baseline to 2 h in response to the cake items (CAKE-FRESH +32%; CAKE-STORED +36%, *P* < 0.001), but not the meat items (MEAT-FRESH: +3%; MEAT-STORED: +2%, *P* > 0.05) (Fig.3). Further, the peak total n-3 PUFA concentration was significantly higher for one of the cake items (CAKE-STORED, 80.9 ± 21.4 *μ*g/mL) versus both of the meat items (MEAT-FRESH, 66.7 ± 18.9 *μ*g/mL and MEAT-STORED, 66.0 ± 16.9 *μ*g/mL) (*P* < 0.05), regardless of postprandial timing; however, differences in peak total n-3 PUFA concentration between CAKE-FRESH and the meat items did not reach statistical significance. Lastly, n-3 PUFA concentrations significantly declined from 4 to 6 h after consumption of CAKE-STORED, while the postprandial response to the meat items was in level during this time period. The apparent decline in n-3 PUFA concentrations from 4 to 6 h (Fig.3), after consumption of CAKE-FRESH, did not reach statistical significance.

Bioavailability of microencapsulated DHA/EPA, within low- and high-protein food items, did not appear to be affected by high-temperature shelf-storage. The slower absorption rate in response to the meat items compared to cake items is not surprising, since protein is digested more slowly than carbohydrates; and, chemical reactions between the gelatin encapsulate (e.g., disulfide bonding and Maillard browning) may strengthen the encapsulate, thus making the encapsulate less susceptible to digestive enzymes (Meade et al. 2005) and slowing digestion. Both food types are acceptable delivery vehicles, but n-3 appearance seems to follow a slower time course when the meat stick is the delivery vehicle.

## Conclusions

Consuming omega-3 fatty acids, particularly DHA and EPA, may have beneficial effects on human health that could warrant fortification of n-3 content of combat rations and other commercial shelf-stable foods. However, incorporating n-3s into shelf-stable food items presents challenges, because they are susceptible to degradation during high-temperature shelf-storage. Microencapsulating DHA and EPA in gelatin, prior to incorporating them into food, protects against degradation of n-3s during storage. We observed no appreciable differences in n-3 bioavailability between food items that were fresh versus those that were previously stored, suggesting that prolonged high-temperature storage is not compromising n-3 absorption. The results indicate that there is a delay in LCn3 and total n-3 PUFA appearance in response to meat items versus cake items. Taken together, it appears that the microencapsulation technology successfully preserved the bioavailability of LCn3, and both the low- and high-protein food items are suitable shelf-stable food items for fortification with encapsulated DHA/EPA.
